# Proposal of supervised data analysis strategy of plasma miRNAs from hybridisation array data with an application to assess hemolysis-related deregulation

**DOI:** 10.1186/s12859-015-0820-9

**Published:** 2015-11-18

**Authors:** Elena Landoni, Rosalba Miceli, Maurizio Callari, Paola Tiberio, Valentina Appierto, Valentina Angeloni, Luigi Mariani, Maria Grazia Daidone

**Affiliations:** Clinical Epidemiology Unit, Fondazione IRCCS Istituto Nazionale dei Tumori, Via Venezian 1, 20133 Milan, Italy; Biomarkers Unit, Fondazione IRCCS Istituto Nazionale dei Tumori, Via Venezian 1, 20133 Milan, Italy

**Keywords:** Data mining, Feature selection, Machine learning, Class prediction, High-dimensional data, SVM, Plasma miRNAs

## Abstract

**Background:**

Plasma miRNAs have the potential as cancer biomarkers but no consolidated guidelines for data mining in this field are available. The purpose of the study was to apply a supervised data analysis strategy in a context where prior knowledge is available, *i.e.*, that of hemolysis-related miRNAs deregulation, so as to compare our results with existing evidence.

**Results:**

We developed a structured strategy with innovative applications of existing bioinformatics methods for supervised analyses including: 1) the combination of two statistical (t- and Anderson-Darling) test results to detect miRNAs with significant fold change or general distributional differences in class comparison, which could reveal hidden differential biological processes worth to be considered for building predictive tools; 2) a bootstrap selection procedure together with machine learning techniques in class prediction to guarantee the transferability of results and explore the interconnections among the selected miRNAs, which is important for highlighting their inherent biological dependences. The strategy was applied to develop a classifier for discriminating between hemolyzed and not hemolyzed plasma samples, defined according to a recently published hemolysis score. We identified five miRNAs with increased expression in hemolyzed plasma samples (miR-486-5p, miR-92a, miR-451, miR-16, miR-22).

**Conclusions:**

We identified four miRNAs previously reported in the literature as hemolysis related together with a new one (miR-22).which needs further investigations. Our findings confirm the validity of the proposed strategy and, in parallel, the hemolysis score capability to be used as pre-analytic hemolysis detector. R codes for implementing the approaches are provided.

**Electronic supplementary material:**

The online version of this article (doi:10.1186/s12859-015-0820-9) contains supplementary material, which is available to authorized users.

## Background

MicroRNAs (miRNAs) are highly conserved single-stranded small RNA molecules (~19–22 nucleotides long) that play a key role in post-transcriptional gene regulation. To date, more than 2600 human miRNAs have been identified (miRBase V21; http://www.mirbase.org/). This class of small RNAs is being widely studied in oncology and a functional implication in cancer development and progression has been demonstrated [1–3]. Recent studies have shown that miRNAs can be released from cells (encapsulated in exosomes and/or bound to proteins and lipoproteins) and enter into the circulation as a consequence of an active release or apoptotic and necrotic cell death [4–7]. As a result of miRNA release from cells, these molecules have also been found in every human body fluid, in a stable form protected from endogenous RNases, thus making plasma miRNA levels well suited for non invasive analysis in patient samples [8, 9]. Independent studies have reported the feasibility of using plasma miRNAs as promising disease biomarkers and, in the context of malignancies, they have shown a potential as molecular tools for detection, prognosis and treatment decision making of various cancers [10, 11]. However, some biological or technical challenges could limit the development of this class of biomarkers [12, 13], thus probably giving an explanation of the poor concordance among inter-study results [14].

In the attempt to develop a multimarker classifier using plasma miRNA data, some issues arising during the discovery process challenge the researchers. Moreover, so far there are no consolidated guidelines for data analysis in this context. This prompted us to develop a structured strategy for supervised analyses with the aim of: (1) in class comparison analysis, detecting differences of miRNA distributions between the two compared classes(2) in class prediction analysis, discovering the top discriminating features, study their associations and interconnections, and developing a ‘robust’ cross validated classifier. In the class comparison step we proposed the combined use of two tests: the *t*-test and the nonparametric Anderson-Darling (AD) test [15]. The former is commonly applied for class comparison being directly related to the fold change (FC), which is taken as a measure of the ‘differential expression’ direction and strength; however, the FC is limited to the exploration of differences between the mean expression values in the two compared classes. On the other hand, the AD test is able to detect more general differences between two classes, which could reveal hidden differential biological processes. In class prediction we set up an assumption-free procedure for the development of a cross validated classifier, after a robust miRNA ordering via bootstrap sampling.

The above approaches were applied to plasma miRNAs determined on a subset of patient samples from a clinical trial series [16]. RNA extracted from these samples was subjected to Agilent miRNA hybridization array. A microarray approach was chosen because it allows reaching a higher throughput than PCR-based assays (even if it is able to analyze only miRNAs already known and annotated in miRBase [17]) and is expected to be advantageous in a discovery phase. Different miRNA microarray platforms, able to measure circulating miRNAs, are commercially available, including GeneChip miRNA Array by Affimetrix, Human miRNA Microarray by Agilent. Among these, we opted for the Agilent system, since it emerged as one of those obtaining the highest performances and is probably the most commonly used. In addition, in a pilot study that we have recently published, the feasibility of using such a platform in miRNA detection also from archival plasma samples was evaluated [18] and we found a very high correlation between technical replicates and a good correlation between different batches. We focused on the comparison between miRNA expression profiles from hemolyzed and not-hemolyzed plasma samples, thus choosing a context where prior knowledge on deregulated miRNAs is available.

## Methods

The R codes implementing the proposed approach are provided as Additional files 1 and 2. The strategy that we developed for data preparation and data analysis is illustrated in Additional file 3: Figure S1. All analyses were performed using R and in particular Bioconductor libraries (http://www.bioconductor.org). The details are reported below.

### Study design

Plasma samples included in the present study come from patients entering a randomized breast cancer prevention Trial [16]. In details, we analyzed a subset of patients from the group of 1476 patients enrolled in the control (not treated) arm of the trial at the Fondazione IRCCS Istituto Nazionale dei Tumori. Blood samples, collected using heparin, were separated into plasma aliquots by centrifugation (2000 × g; 15 min at 4 °C) and stored at −80 °C until assayed; no thawing accident occurred during storage. Since the blood samples were collected for different purposes, no information are available on erythrocyte or platelet counts. Nevertheless, the presence of hemolysis was evaluated in the plasma samples on the basis of the ‘Hemolysis Score’ (HS) previously published by our group [19]. Our ‘controls’ were not-hemolyzed plasma samples (HS ≤ 0.057) and our ‘cases’ were the samples with HS > 0.14, roughly corresponding to a visible hemolysis. The remaining samples showing 0.057 < HS ≤ 0.14 were not analyzed. As cases and controls could be unbalanced for some variables, a matching procedure was used, by applying the nearest neighbor matching within specified propensity score (PS) calipers [20] in order to have a more relaxed criterion which would enable us to match all the hemolyzed samples. Given the PS, that is the probability of assignment to one group conditional on some characteristics of patients and samples (*i.e.*, disease status, age at drawing and drawing year), we matched each case with two controls with the closest PS within the specified range (the caliper width). We used the recommended caliper width, which is equal to the 20 % of the standard deviation of the PS logit [21]. After matching we randomly split the sample in half into a training set for supervised analyses and a validation set for internal validation of results, maintaining the 1:2 ratio between cases and controls.

### Sample processing

Plasma isolation and RNA extraction were carried out as previously described [18]. Briefly, total RNA was extracted from 350 μl plasma collected in heparin using the commercial column-based system Qiagen miRNeasy R Mini Kit (Qiagen, Valencia, CA, USA), slightly modified. Briefly, 400 μl of plasma/medium were thawed on ice and centrifuged at 1000 × g for 5 min in a 4 °C microcentrifuge. An aliquot of 350 μl of plasma per sample was transferred into a new microcentrifuge tube and 1300 μl of a Qiazol mixture containing 1.25 μg/ml of MS2 bacteriophage RNA (Roche Applied Science, Milan, Italy) and a RNA spike-in (ath-miR-159a) to be able to eventually test the recovery efficiency by RT-PCR anlysis. A rinse step (500 μl Qiagen RPE buffer) was repeated 3 times. Total RNA was eluted by adding 25 μl of RNase-free water to the membrane of the spin column and incubating for 1 min before centrifugation at 15,000 × g for 1 min at room temperature. The heparin contained in the RNA samples was digested using heparinaseI (Sigma- Aldrich, St. Louis, MO, USA), in the presence of an RNase inhibitor, (RNAsin; Promega, Madison, WI, USA) for 1 h at room temperature, and RNA was stored at −80 °C. The heparinease digestion was performed to make RNA suitable for downstream RT-PCR analysis (not pertinent to this paper, manuscript in preparation). In fact, For many years, the use of heparin for blood collection has been avoided in case of subsequent RNA extraction, since the anticoagulant inhibits PCR amplification [22–25]. However, we have recently demonstrated that if adequately treated with heparinase, plasma samples derived from blood collected with heparin tubes are suitable for miRNA expression analysis, without affecting miRNA detection [26]. Hybridization on Agilent Human miRNA microarrays was carried out by Functional Genomics facility according to the manufacturer’s instructions as previously described [18]. Briey, SurePrint G3 Human v16 miRNA 8x60K microarrays (G4870A) designed on miRBase 16.0 from Agilent Technologies were used. 2.5 μl of total RNA was dephosphorylated at 37 °C for 30 min with calf intestinal phosphatase and denatured using 100 % DMSO at 100 °C for 5 min. Samples were labeled with pCp-Cy3 using T4 ligase by incubation at 16 °C for 1 h and hybridized. Arrays were washed according to manufacturer’s instructions and scanned at a resolution of 5 μm using an Agilent 4000B scanner. Data were acquired using Agilent Feature Extraction software version 10.7.

### Data pre-processing

Raw data were summarized as previously described [18]. Briefly, in the employed platform, each miRNA is targeted by one to four different probes and each probes spotted 10–40 times on the array. Then, the total signal for each miRNA was obtained by summing the probe signals derived from Agilent Feature Extraction software. Using this software, each probe is defined detected if its value is greater than three times its standard error, and each miRNA is defined as detected if at least one of the probes is detected. Summarized data were log_2_ transformed. Only the 1205 human (‘hsa’) miRNAs were considered in subsequent analyses. Microarray data are MIAME compliant and were deposited into the NCBI’s Gene Expression Omnibus (GEO) database with accession number ‘GSE59993’ (http://www.ncbi.nlm.nih.gov/geo/). MiRNAs were filtered at 90 %, *i.e.*, we retained only miRNAs detected in at least 90 % of all samples. By applying a less stringent filtering (*i.e.*, 10 % filtering), no additional differentially expressed (DE) miRNAs could be identified (data not shown), as compared with those obtained with the 90 % filtering.

As regard to the normalization step, we applied the ratio-based approach [27] that is like using, in turn, all miRNAs as normalizers but eliminating any duplications, *i.e.*, each miRNA pair only appeared once.

### Supervised data analyses

We implemented supervised approaches for class comparison and class prediction on the training set samples using both raw (not normalized) and ratio-normalized data. Class comparison analysis, aimed at identifying features (miRNAs or miRNA ratios) DE between cases and controls, was based on the combined use of the t- and the non parametric AD [15] tests. While the *t*-test considers only location differences, the AD test is an ‘omnibus test’ [28], *i.e.*, it considers the whole feature distribution, granting more importance to the observations in the tails. The latter characteristic becomes valuable when one is interested in finding signals that are only present in patient subsets diverging from the center of the distribution.

Moreover, plasma miRNA data, like other ‘omics’ data, have often not normal distributions and the sample sizes are often small. In presence of distributions with asymmetries, multimodality or heavy tails, the AD test reveals useful for the identification of interesting features. We considered the asymptotic version of the AD test, with correction for the presence of ties. The Benjamini-Hochberg method was used to distinctly adjust t- and AD p-values in order to control for the False Discovery Rate (FDR) [29]. In particular, we combined the results of the two tests by considering as significantly DE the features for which the FDR-adjusted p-value was below the 5 % level for at least one of the two tests. This procedure could inflate the overall Type I error; however, we expect such an effect to be marginal because the two tests statistics are likely to be dependent and, in addition, both tests are applied to the same data.

For class prediction analysis, aimed at developing a classifier able to accurately discriminate between hemolyzed and not-hemolyzed samples, a two-step procedure was set up: firstly, with the purpose of obtaining a robust ranking of features with distributional differences between the two classes, a ‘bootstrap selection’ was performed, according to the strategy proposed by Austin and Tu [30]. We extracted 1000 bootstrap samples [31] and we applied three machine learning selection algorithms, *i.e.*, Prediction Analysis for Microarrays (PAM) [32], Random Forests (RF) with Boruta feature selection method [33] and Elastic Smoothly Clipped Absolute Deviation (SCAD) Support Vector Machines (SVM) [34], while maintaining the same proportion of hemolyzed and not-hemolyzed in each group. The three methods were chosen because they overcome the ‘curse of dimensionality’ usually present in high-dimensional data (*i.e.*, more features than subjects) and because they are conceptually different algorithms that we considered as ‘representative’ of methodological categories using different decision rules for classification (*i.e.*, a nearest centroid, a decision tree and a SVM based method, respectively). PAM, being characterized by a minor complexity respect to the other two algorithms, may be insufficient to appreciate complex classification patterns. Among the other two more sophisticated methods, RF overcome the main disadvantage of decision trees methods, which is their tendency to data overfitting and, like PAM, are fast and nonparametric, so one has not to worry about outliers. On the other hand, RF only output measures of feature importance, the interpretation of which is controversial with correlated features [35]. The inherent biological dependence among the features, which implies correlation among miRNAs, was taken into account by using the Elastic SCAD SVM algorithm. The features were ranked on the basis of the frequency of simultaneous selection by the three above algorithms, discarding the features not selected in at least one bootstrap sample. None of the three algorithms is uniformly superior in detecting class differences. Our strategy seeks to overcome the above limitation by implicitly relying on an intersection criterion, by which a feature emerges as ‘strong’ regardless of the statistical technique used for analysis. As second step, aimed at developing a cross validated classifier, we implemented a linear SVM model [36], using the features previously ranked according to the bootstrap selection. We chose the linear SVM since it is a simple model requiring only the tuning of two parameters, *i.e.*, the cost, which controls model complexity and the class weights, indicating the influence assigned to the two classes. Different models were fitted by varying the number of included features, forwardly selected according to the bootstrap generated list. The models were then cross validated with a leave-one-out cross validation procedure [37] to adjust for overoptimism the classification performance measures,* i.e.*, sensitivity, specificity and Youden index [38]. The final model used for developing the classifier was chosen according to both the criteria: best classification performance, measured by the highest Youden index, and smallest number of features included in the model. Finally, the classification performance measures of the chosen models were calculated on the validation set, together with their corresponding bootstrap 95 % confidence intervals (CI) taken as an estimate of the performance measure variability.

## Results and discussion

### Sample processing and data pre-processing

After case–control matching, 78 samples were selected, 26 hemolyzed and 52 not-hemolyzed; 39 samples (13 hemolyzed vs 26 not-hemolyzed) were included in the training and validation set, respectively. The summary distribution of matching variables before and after matching is included in the Additional file 4: Table S1. After the filtering performed on the training set, 88 miRNAs were retained, based on which a total of 3828 ratios were generated.

### Class comparison

Additional file 5: Table S2 shows the results of class comparison using raw and ratio data with the lists of miRNAs significantly DE according to t- or AD test, after adjusting for multiple testing. The same results were graphically summarized via volcano and concordance plots (Additional file 6: Figure S2). Concerning raw data, four miRNAs (4.5 %) were significant at the t- or AD test. Three miRNAs (miR-486-5p, miR-92a, miR-451) were identified as up-regulated in hemolyzed samples through the *t*-test (Additional file 6: Figure S2A), being also detected by the AD test, as shown in the second quadrant of the concordance plot in the Additional file 6: Figure S2B (the adjusted p-values were coincident). Moreover, one more miRNA (miR-16) was significant according to the AD test alone (Additional file 6: Figure S2B), although the *t*-test p-value was near to the significance threshold. Regarding ratio data, 224 miRNA ratios (5.8 %) were significant at the t- or AD test. We detected 104 ratios as significantly up-regulated and 94 ratios as significantly down-regulated with the *t*-test, for a total of 198 ratios, which involved 80 miRNAs (Additional file 6: Figure S2C). One hundred and seventy ratios (involving 68 miRNAs, including the four previously selected with raw data) were detected by both tests (first quadrant of Additional file 6: Figure S2D), 28 ratios (involving 27 miRNAs) only by the *t*-test (second quadrant of Additional file 6: Figure S2D) and 26 ratios (involving 29 miRNAs) only by the AD test (fourth quadrant of Additional file 6: Figure S2D). The features significantly DE in the training set at the raw and ratio data analysis were also evaluated in the validation set. All the 4 miRNAs and 203 over 224 ratios resulted DE in the validation set for the t- or the AD test (Additional file 5: Table S2).

### Class prediction

**Fig. 1:**
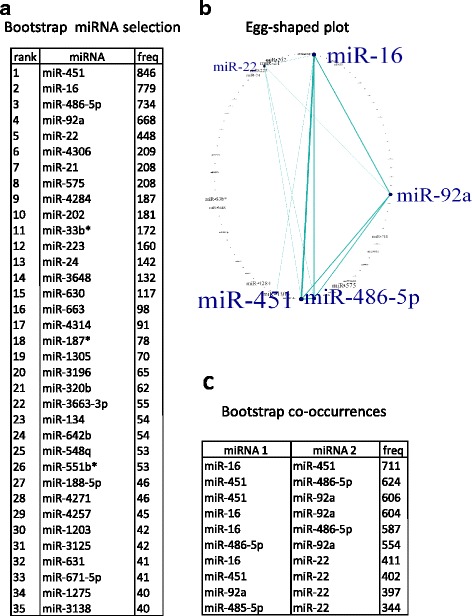
Results of the first step of class prediction performed in the training set raw data. **a** Bootstrap occurrences of the top 35 miRNAs included in the chosen model. **b** Egg-shaped plot. Node size and line thickness are proportional to the frequency of bootstrap occurrences and co-occurrences, respectively. A filter was applied to show only the features with at least 300 co-occurrences. **c** Bootstrap co-occurrences of the most interconnected miRNAs

The complete lists of the bootstrap-ranked features for both raw and ratio data are reported in the Additional file 7: Table S3. Figure 1 summarizes the results of the first step of class prediction analysis with raw data (‘bootstrap selection’). In particular, in Fig. 1a the miRNAs are ranked according to the number N of occurrences in the bootstrap samples, *i.e.*, the number of times in which they are jointly selected by the three machine learning algorithms. miRNAs identified in class comparison analysis as significantly up-regulated in hemolyzed samples resulted at the top positions of bootstrap ranking (top 35 miRNAs in Fig. 1a). MiR-451 headed clearly in class prediction, being selected in 846 out of 1000 bootstrap samples, followed by miR-16 (779/1000), miR-486-5p (734/1000), miR-92a (668/1000) and miR-22 (448/1000). An egg-shaped plot representation of top ranking miRNAs is shown in Fig. 1b, where node size and edge thickness are proportional to the frequency of miRNAs occurrences and co-occurrences (pairwise occurrences) in the bootstrap samples; a filtering was applied to show only those miRNAs with co-occurrences at least equal to 300. The most frequent co-occurrences are shown in Fig. 1c. Generally, the most selected miRNAs were also the most interconnected. In fact, considering miR-451, the strongest co-occurrence involved miR-16, being the two miRNAs jointly selected in 711 out of 1000 bootstrap samples, followed by miR-486-5p (624 co-occurrences) and miR-92a (606 co-occurrences). Also miR-16 presented several interconnections with miR-92a (604 co-occurrences), miR-486-5p (587 co-occurrences), and miR-22 (411 co-occurrences). MiR-451, miR-16, miR-486-5p and miR-92a have been previously reported in the literature as hemolysis-related plasma miRNAs (http://www.bioconductor.org), while miR-22 was selected in a high number of bootstrap samples and linked to the top four miRNAs. Ratio data generally led to smaller bootstrap occurrences, since each miRNA appeared in several ratios. However, miR-486-5p, miR-92a, miR-451 and miR-16 were included in the top eight ratios, with occurrences equal to 357 (1^st^ position), 304 (2^nd^ position), 270 (4^th^ position) and 214 (8^th^ position), respectively. MiR-22 appeared at the 31^st^ position, with 121 occurrences. The ‘autoselected’ specific normalizers were miR-4257 for miR-486-5p and miR-92a, and miR-4286 for miR-451 and miR-16. The top co-occurrence involved miR-92a/miR-4257 and miR-486-5p/miR-4257, with a frequency equal to 200.

**Fig. 2:**
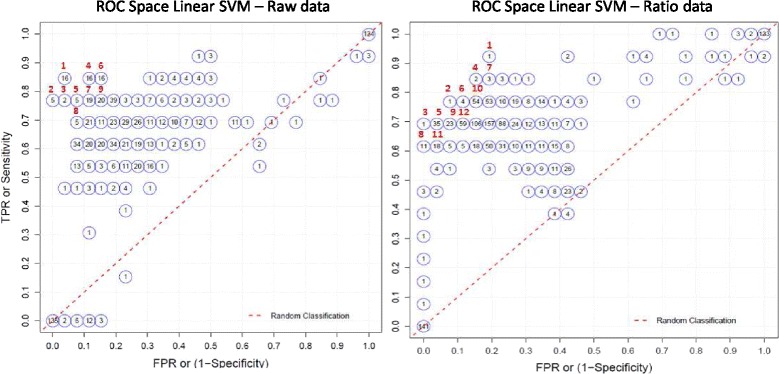
Results of the second step of class prediction performed in the training set raw andratio data. ‘ROC space’ plot representing the classification performance of different models for class prediction in terms of false positive rate (FPR) and true positive rate (TPR) in the training set raw data (left panel) and ratio data (right panel). As true for the ROC curves, ideal models are those closest to the point (0,1), corresponding to 100 % sensitivity and specificity.

As regard to the classifier development (step 2), the ‘ROC space’ plot in Fig. 2 summarizes the SVM model performance in terms of false positive rate (FPR) and true positive rate (TPR); as true for the ROC curves, ideal models are those closest to the point (0,1), corresponding to 100 % sensitivity and specificity. The numbers inside the circles count the models with a specific combination of FPR and TPR, while the numbers outside (ID) rank each group of models in terms of performance, as quantified by the Youden index (*e.g.*, ID = 1 indicates the group of models with the highest Youden index). Considering raw data (Fig. 2a), we identified 8 best performing groups; among them, 16 models (ID = 1) showed the highest Youden index equal to 0.81. Using ratio data (Fig. 2b) only one model stood alone in leading the rank classification list, with a Youden index of 0.73.

The above results are numerically shown in Table 1 (left panel) only for the best performing groups, *i.e.*, those ID numbered in the Fig. 2; additionally, for the specific model chosen in each group according to a parsimony criterion (smallest number of features), we show the parameters (middle panel) and the performance evaluated in the validation set (right panel). Considering raw data, the Youden index ranged from 0.61 to 0.81 in the training set and from 0.46 to 0.73 in the validation set. Among the 16 models with ID = 1 (Youden index = 0.81), the chosen one included 35 miRNAs (Fig. 1a). However, an alternative choice could be the one selected within the ID = 8 group (Youden index = 0.61), which included three miRNAs, *i.e.*, miR-451, miR-16 and miR-486-5p; such a model achieved the highest classification performance in the validation set (Youden index = 0.73).

**Table 1:** Model classification performance measures in the training and validation sets with raw and ratio data.

Training set								Validation set		
Classication performance of the best performing groups of models					Parameters of the chosen model			Classication performance of the chosen model		
Group ID	N models	Sens	Spec	Youden index	N miR	SVM cost	SVM weights	Sens [CI]	Spec [CI]	Youden index [CI]
1	16	0.85	0.96	0.81	35	10	(0.5; 0.5)	0.77 [0.54–0.92]	0.77 [0.61–0.92]	0.54 [0.23–0.81]
2	5	0.77	1.00	0.77	35	1	(0.5; 0.5)	0.85 [0.61–1.00]	0.81 [0.65–0.92]	0.65 [0.38–0.85]
3	2	0.77	0.96	0.73	30	1	(0.5; 0.5)	0.85 [0.61–1.00]	0.85 [0.69–0.96]	0.69 [0.42–0.88]
4	16	0.85	0.88	0.73	40	10	(0.5; 0.5)	0.77 [0.54–0.92]	0.73 [0.54–0.88	0.50 [0.19–0.77]
5	5	0.77	0.92	0.69	35	1	(0.4; 0.6)	0.85 [0.61–1.00]	0.73 [0.54–0.88]	0.58 [0.31–0.81]
6	16	0.85	0.85	0.69	50	10	(0.5; 0.5)	0.85 [0.61–1.00	0.69 [0.50–0.88	0.54 [0.27–0.81]
7	19	0.77	0.88	0.65	40	1	(0.4; 0.6)	0.77 [0.54–0.92]	0.69 [0.50–0.85]	0.46 [0.15–0.73]
8	5	0.69	0.92	0.61	3	100	(0.4; 0.6)	0.77 [0.54–0.92]	0.96 [0.88–1.00]	0.73 [0.46–0.92]
9	20	0.77	0.85	0.61	5	10	(0.4; 0.6)	0.69 [0.46–0.92]	0.92 [0.81–1.00]	0.61 [0.35–0.85]
**Ratio data**										
1	1	0.92	0.81	0.73	500 (88)	0.01	(0.2; 0.8)	0.92 [0.77–1.00]	0.65 [0.46–0.85]	0.58 [0.31–0.81]
2	1	0.77	0.92	0.69	17 (16)	0.01	(0.3; 0.7)	0.77 [0.54–0.92]	0.92 [0.81–1.00]	0.69 [0.42–0.92]
3	1	0.69	1.00	0.69	90 (50)	0.01	(0.5; 0.5)	0.69 [0.38–0.92]	1.00 [1.00–1.00]	0.69 [0.38–0.92]
4	2	0.85	0.85	0.69	150 (66)	0.01	(0.2; 0.8)	0.92 [0.77–1.00]	0.69 [0.50–0.85]	0.61 [0.38–0.81]
5	35	0.69	0.96	0.65	4 (5)	0.1	(0.5; 0.5)	0.77 [0.54–0.92]	1.00 [1.00–1.00]	0.77 [0.54–0.92]
6	4	0.77	0.88	0.65	500 (88)	0.01	(0.4; 0.6)	0.92 [0.77–1.00]	0.77 [0.58–0.92]	0.69 [0.46–0.88]
7	3	0.85	0.81	0.65	600 (88)	0.01	(0.2; 0.8)	0.92 [0.77–1.00]	0.65 [0.46–0.85]	0.58 [0.31–0.81]
8	11	0.61	1.00	0.61	2 (3)	0.1	(0.5; 0.5)	0.77 [0.54–0.92]	1.00 [1.00–1.00]	0.77 [0.54–0.92]
9	23	0.69	0.92	0.61	3 (4)	0.1	(0.4; 0.6)	0.77 [0.54–0.92]	0.96 [0.88–1.00]	0.73 [0.50–0.92]
10	54	0.77	0.85	0.61	4 (5)	0.1	(0.3; 0.7)	0.77 [0.54–0.92]	0.88 [0.73–1.00]	0.65 [0.38–0.88]
11	18	0.61	0.96	0.58	3 (4)	0.1	(0.5; 0.5)	0.77 [0.54–0.92]	1.00 [1.00–1.00]	0.77 [0.54–0.92]
12	59	0.69	0.88	0.58	2 (3)	10	(0.4; 0.6)	0.77 [0.54–0.92]	1.00 [1.00–1.00]	0.77 [0.54–0.92]

In the last three columns, validation set classification performance measures are reported together with the corresponding bootstrap 95 % confidence intevals (CI)

*Abbreviations*: *Group ID* ID of the groups of best performing models (see also Fig. 2); *N models* number of models in each group, showing a specific classification performance, *Sens* sensitivity, *Spec* specificity, *N miR* number of miRNAs included in the model chosen in each group for containing the smallest number of miRNAs, *SVM cost* cost parameter of the linear SVM model, *SVM weights* weight parameter of the linear SVM model

Regarding ratio data, the Youden index ranged from 0.61 to 0.73 in the training set and from 0.58 to 0.77 in the validation set. The chosen model included 500 ratios (Youden index = 0.73), corresponding to 88 features. Alternative choices could be the model with ID = 8, including two ratios (miR-486-5p/miR-4257 and miR-92a/miR-4257) or that with ID = 5, including 4 ratios (miR-486-5p/miR-4257, miR-92a/miR-4257, miR-486-5p/miR-4286, miR-4286/miR-451), the latter presenting a slightly better classification performance in the training set (Youden index = 0.65 vs 0.61); also in this case, the two parsimonious models had the best performance in the validation set (Youden index = 0.77). It is worth to notice that with ratio data the miR-16 would not have been selected, since the top ratios contained more than once the other hemolysis-related miRNAs, producing redundancy in the results.

Globally, we noticed that the SVM cost parameters, which control model complexity, were smaller with the ratio data and that, regardless the type of data, it was more difficult to validate a model containing a large number of miRNAs. Moreover, in the validation set the Youden index showed wide bootstrap confidence intervals (CI), due to the small sample size.

## Conclusions

In the present work we developed a general analysis strategy in order to deal with some issues arising in the supervised analyses of plasma miRNA from hybridisation array data. In the data pre-processing step, any normalization method can be applied and does not preclude the subsequent conduction of supervised analyses, although contributing to the final results. The normalization method should be chosen in relation to the type of features, their precision level and to the domain knowledge (*e.g.*, possible availability of housekeeping features). While in our investigation we adopted a joint analysis of raw and ratio-normalized data, other methods might be suitable, like for instance the quantile method, previously shown to work best in reducing differences in miRNA expression values for tissue samples [39]. We just considered inappropriate the application of the global mean method, which would artificially produce down-regulated miRNAs. Such a problem was clearly demonstrated in the case of an expected general miRNA down-regulation as a consequence of inducible deletion of Dicer1 [40]. This is in contrast with the expectation of a global miRNA up-regulation in patients with cancer as a consequence of a passive (*i.e.*, cancer cell death) or active (*i.e.*, by microvesicles) release in bloodstream. To establish which miRNA in a ratio has relevant discriminating role and which act as normalizer (no modulation, *i.e.*, FC = 1, or presenting weaker modulation) the results of raw and ratio data analyses should be interpreted together. An advantage of the ratio method is that, in the absence of known housekeeping miRNAs, it allows identification and automatic handling of a specific normalizer for each DE miRNA.

In class comparison analysis, the search for DE features is usually intended for detecting significantly different means in the two groups, and location tests, such as the *t*-test, are commonly applied; this classifies class comparison analysis in the domain of univariable statistical analyses. However, the *t*-test assumption of normality is often not fulfilled when dealing with plasma miRNA data, mainly due to the skewed, heavy-tailed or multimodal distributions of expression values, especially if associated with small sample size. Moreover, focusing only on location, the *t*-test could miss miRNAs with a signal translating into more general differences between the distributions. Our strategy of combining the results of t- and AD tests was aimed at taking advantage of their different characteristics and allowed us to discover those miRNAs discarded by the *t*-test due to not significant FC, but with not overlapping feature distributions. The AD test is particularly valuable when distributions differ in the tails, which could reveal underlying biological differences. Class comparison analysis is a useful tool for detecting DE features; however, in our opinion caution should be taken in using it for ranking purposes. Indeed, by using the bootstrap selection in the first step of class prediction analysis, together with the application of the three machine learning algorithms (Elastic SCAD SVM, RF, PAM), more robust and possibly generalizable results can be obtained. Together with the bootstrap selection, we want to point out the egg-shaped plot, which can be used as a tool for giving an insight of interconnections among the selected features, becoming useful for highlighting their inherent biological dependences.

In the second step of the class prediction analysis, the classifiers are obtained by using statistical models including subgroups of selected features, and this categorizes class prediction in the domain of multivariable statistical analyses. The joint use of bootstrap selection and classifier cross validation should ensure the robustness of the class prediction results. A limitation of the procedure is that we could identify several best models in terms of classification performance. In some cases (especially using ratio data) the best models included a large number of features, thus being more prone to overfitting. However, we observed that the use of a small number of strongly predictive features resulted in a non significant decay of the cross validated classification performance measures in the testing set. Therefore, our strategy was to choose more parsimonious models, since is likely that the features included in such models will not be filtered out during the data pre-processing step. However, our results have to be taken with caution due to the small sample size, as it emerged from the large bootstrap intervals of the classification performance measures. By using our strategy we identified four top miRNAs (miR-486-5p, miR-92a, miR-451, miR-16) that have been reported in the literature as related to the presence of hemolysis, together with another one (miR-22), which is worth to further investigate. Even though miR-22 was not directly described as hemolyis-susceptible miRNA, it was identified as a signature miRNA for erythrocyte maturation [41]. In addition, very recently MacLellan et al., by mimicking hemolysis through mechanical lysis of blood samples in healthy individuals, found higher levels of serum miR-22 in lysed compared to matched unlysed samples ([42], Fig. 1). Regarding the top miRNAs, we obtained consistent results in class comparison and bootstrap selection; indeed, strong signals are detectable on both raw and ratio data, even with univariable and not cross validated analyses. However, univariable methods unavoidably discard features that would have provided useful information, if taken in aggregate. More subtle differences, like those we observed for miR-22, could justify the use of more sophisticated methods, such as the bootstrap selection joined with the machine learning algorithms. The concordance of our results with literature data also corroborated the ability of the HS to discriminate between hemolyzed and not-hemolyzed samples and thus its usefulness as a pre-analytic hemolysis detector.

Classifier development should rely on availability of three distinct datasets for training, validation, and testing. We are aware that a limitation of the present study is the lack of availability of a testing set on which an unbiased assessment of classifier performance could be obtained. Unfortunately, threefold splitting was not applicable in our case study, because was hampered by the small number of hemolyzed samples, and suitable public datasets (*i.e.*, data from Agilent miRNA hybridization array coupled with hemolysis score evaluation) were still unavailable.

Our strategy may be extended to other kinds of ‘omics’ studies by introducing proper methodological adjustments. For instance, with non-coding RNA Sequencing data, which are count variables, the Anderson-Darling test could be used for class comparison analysis; in class prediction analysis, models suitable for analyzing count data should be used (*i.e.*, Negative Binomial, Poisson distribution based models).

To conclude, in this study we implemented a global strategy for the analysis of plasma miRNAs. In class comparison the combination of the results of the t- and the AD tests can be considered valuable to detect miRNAs with significant FC or more general distributional differences between classes, which could reveal hidden differential biological processes worth to be considered for building predictive tools. The use of robust miRNA selection procedure together with multivariable modeling as a strategy employed in class prediction can guarantee result generalizability and be useful to explore the interconnections among the selected miRNAs, which are essential for highlighting their inherent biological dependences.

### Ethics statement

All patients whose blood samples were included in the study signed an informed consent, approved by the Independent Ethical Committee of the Fondazione IRCCS Istituto Nazionale dei Tumori Milano (INT) that approved the use of the samples for this specific study and the relative data publication.

## Additional files

Additional file 1:
**R codes for implementing the described analyses (sample processing, data pre-processing, class comparison and class prediction).** Caliper matching was implemented using the nonrandom package; the t- and the AD tests were implemented using the stats package and the *adk* package, respectively. Notice that the updated package for implementing the AD test is *kSamples*. As regards the bootstrap selection and the egg-shaped plot, we respectively modified the *doBS* and the importance *igraph* functions, both included in the *bootfs* package. For the SVM model we used the *e1071* package. (R 12 kb) Additional file 2:
**R codes for user-defined functions.** (R 15 kb) Additional file 3: Figure S1. Workflow of the strategy used for sample processing, data pre-processing and supervised data analyses. (TIF 148 kb) Additional file 4: Table S1. Summary distribution of matching variables before and after matching. (XLS 22 kb) Additional file 5: Table S2. Class comparison results in the training set with raw and ratio data. List of miRNAs and miRNA ratios significantly differentially expressed according to t- or AD test. The table also shows the class comparison results obtained on the validation set for the same miRNAs and miRNA ratios. Abbreviations: 'med': median of miRNA expression values; 'FC': miRNA fold change; 't.p': p-value at *t*-test; 'AD.p': p-value at AD test; 't.pFDR': False Discovery Rate (FDR)-adjusted p-value at *t*-test; 'AD.pFDR': FDR-adjusted p-value at AD test. (XLS 2059 kb) Additional file 6: Figure S2. Class comparison results in the training set with raw and ratio data. *t*-test volcano plots and concordance plots between t- and Anderson-Darling (AD) test for raw data (panels **A** and **B**) and ratio data (panels **C** and **D**). In the volcano plots the log_2_ feature fold change is plotted on the x-axis and the negative log_10_ p-value at *t*-test is plotted on the y-axis. The horizontal line indicates the 5 % significance level, while n is the number of significantly up-regulated (first quadrant) and down-regulated (second quadrant) features. In the concordance plots the negative log_10_ p-value according to the AD test is plotted on the x-axis and the negative log_10_ p-value according to the *t*-test is plotted on the y-axis. Points lying on the dashed line would indicate perfect concordance between the two tests. (TIF 114 kb) Additional file 7: Table S3. Complete list of features selected in the training set with raw and ratio data by applying Elastic Smoothly Clipped Absolute Deviation (SCAD) Support Vector Machines (SVM), Random Forests (RF) with Boruta feature selection method and Prediction Analysis for Microarrays (PAM) on bootstrap samples. (XLS 114 kb)
