# Supplementary figures and images for: Proposal of supervised data analysis strategy of plasma miRNAs from hybridisation array data with an application to assess hemolysis-related deregulation
Source: BMC Bioinformatics. 2015 Nov 18;16:388. doi: 10.1186/s12859-015-0820-9 (PMC4650369; doi:10.1186/s12859-015-0820-9)

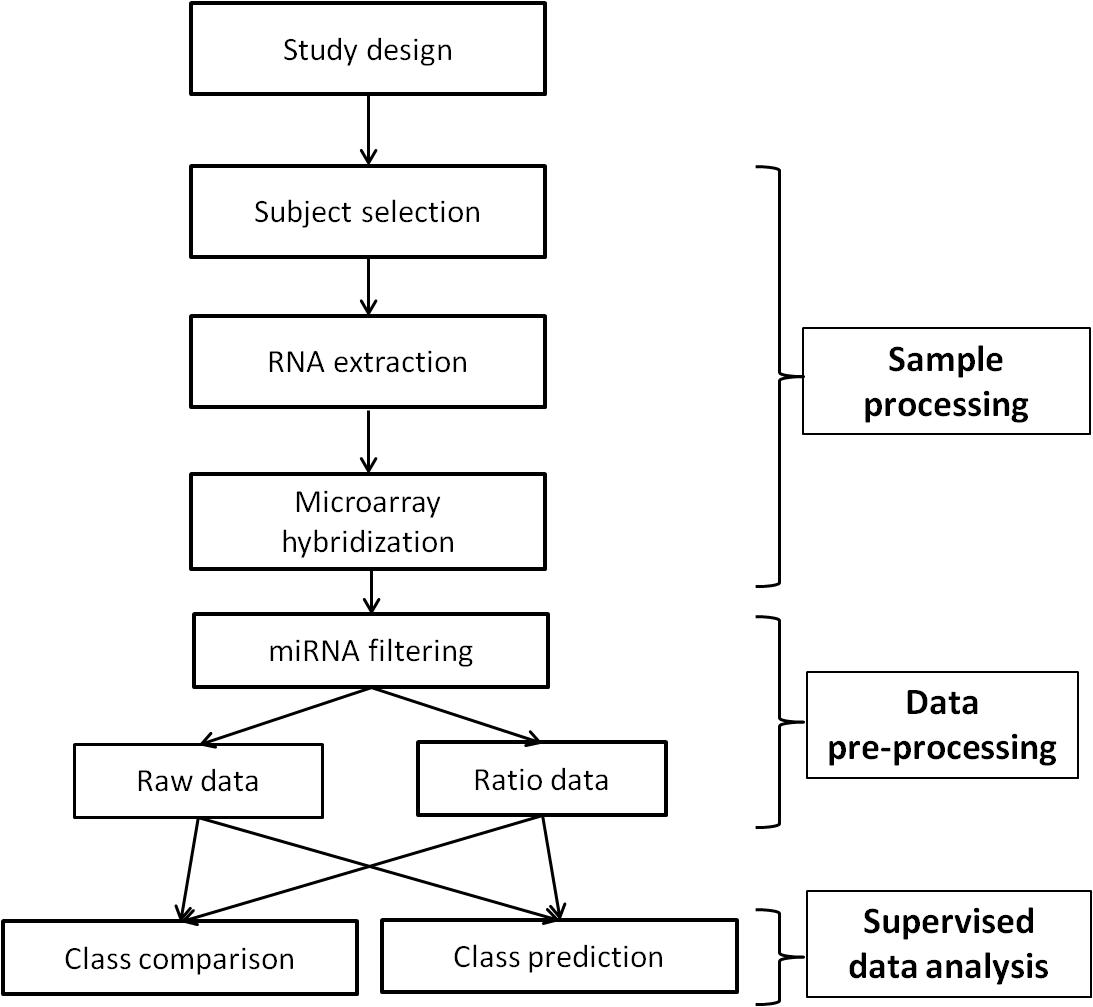

Supplement: Additional file 3: Figure S1. — Workflow of the strategy used for sample processing, data pre-processing and supervised data analyses. (TIF 148 kb) [file 12859_2015_820_MOESM3_ESM.tif]

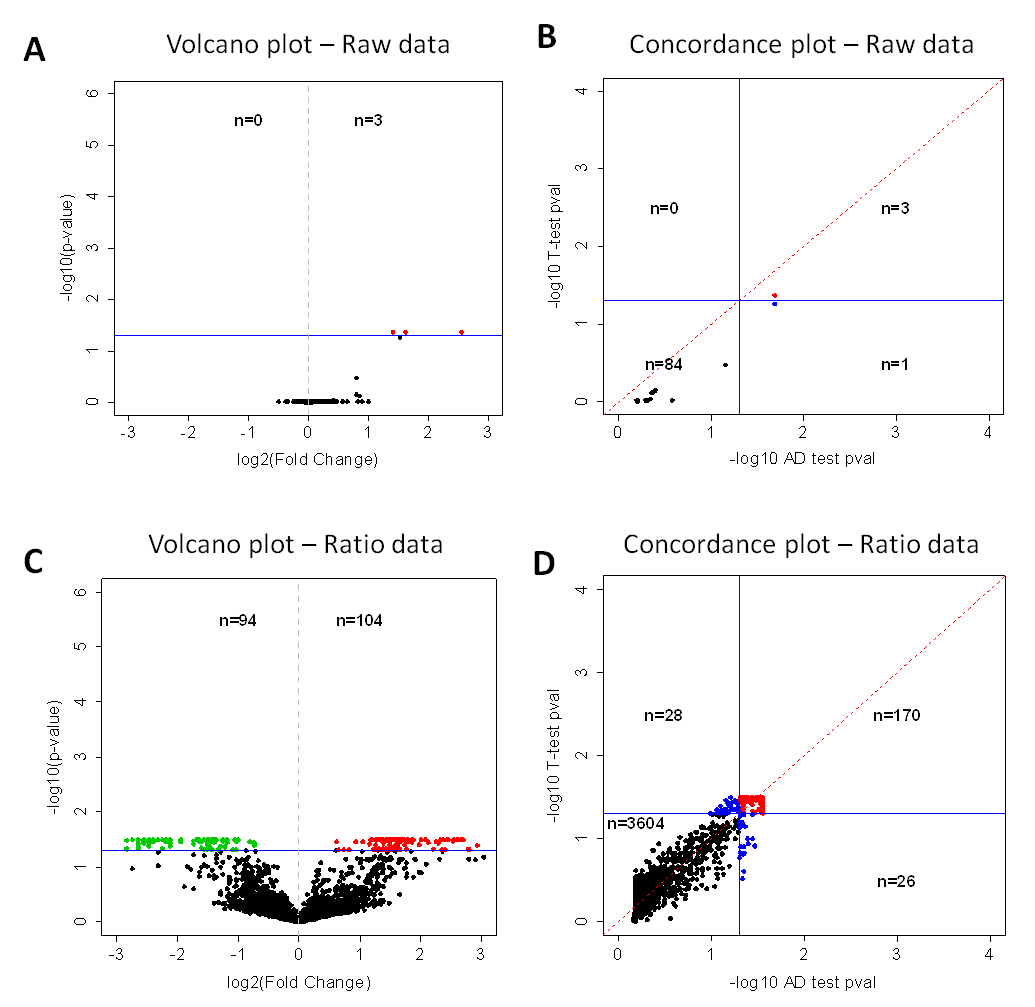

Supplement: Additional file 6: Figure S2. — Class comparison results in the training set with raw and ratio data. t-test volcano plots and concordance plots between t- and Anderson-Darling (AD) test for raw data (panels A and B) and ratio data (panels C and D). In the volcano plots the log2 feature fold change is plotted on the x-axis and the negative log10 p-value at t-test is plotted on the y-axis. The horizontal line indicates the 5 % significance level, while n is the number of significantly up-regulated (first quadrant) and down-regulated (second quadrant) features. In the concordance plots the negative log10 p-value according to the AD test is plotted on the x-axis and the negative log10 p-value according to the t-test is plotted on the y-axis. Points lying on the dashed line would indicate perfect concordance between the two tests. (TIF 114 kb) [file 12859_2015_820_MOESM6_ESM.tif]
